# Delirium could be an indicator of sepsis in patients under 65 years old with urinary tract infections

**DOI:** 10.1186/cc10943

**Published:** 2012-03-20

**Authors:** U Yamada, K Yokota, D Ohta, K Furukawa

**Affiliations:** 1 St Luke's International Hospital, Tokyo, Japan

## Introduction

Delirium, known as sepsis-associated encephalopathy, is a frequent complication of sepsis and may be an independent predictor of mortality of septic patients [1]. A recent study reported delirium could be a predictor or an early marker of sepsis in CABG patients [2]. Urinary tract infection (UTI) often complicates sepsis and delirium; however, relations between delirium and sepsis in UTI patients have not been well investigated. We assessed the relationship between delirium and sepsis in patients with UTI.

## Methods

This study was conducted at St Luke's International Hospital in Tokyo, Japan between January 2009 and October 2011. UTI and sepsis were diagnosed based on positive bacterial cultures and clinical symptoms. Delirium was screened with the Delirium Screening Tool (the 11-item questionnaire, sensitivity 98% and specificity 76%) by trained physicians and nurses. Medical records of patients were reviewed to collect information including age, sex and complications. The association between possible risk factors and delirium was analyzed by chi-squared tests and *t *tests. Statistical analysis was performed using SPSS software version 15.0J.

## Results

Of all 1,727 UTI patients, 905 were men and the mean age was 73.65 ± 14.1. In total, 425 patients (24.6%) became delirious, and 247 patients (14.3%) had sepsis. There was no significant association between sepsis and delirium (*P *= 0.051). However, in the younger population (age <65) delirium occurred significantly more frequently in septic patients than in nonseptic patients (22.9% vs. 10%, *P *< 0.001).

## Conclusion

Among UTI patients, sepsis may increase the complication of delirium. Especially in patients under 65 years old with UTI, delirium symptoms can be a marker for complication of sepsis. In contrast, delirium of patients aged 65 or over could be associated with not only sepsis but also other factors such as dementia, aging and UTI itself.
